# Status of patient safety culture in Arab countries: a systematic review

**DOI:** 10.1136/bmjopen-2016-013487

**Published:** 2017-02-24

**Authors:** Mustafa Elmontsri, Ahmed Almashrafi, Ricky Banarsee, Azeem Majeed

**Affiliations:** Department of Primary Care and Public Health, School of Public Health, Imperial College London, London, UK

**Keywords:** Patient Safety, Safety Culture, Arab Countries

## Abstract

**Objectives:**

To explore the status of patient safety culture in Arab countries based on the findings of the Hospital Survey on Patient Safety Culture (HSPSC).

**Design:**

Systematic review.

**Methods:**

We performed electronic searches of the MEDLINE, EMBASE, CINAHL, ProQuest and PsychINFO, Google Scholar and PubMed databases, with manual searches of bibliographies of included articles and key journals. We included studies that were conducted in the Arab countries that were focused on patient safety culture. 2 reviewers independently verified that the studies met the inclusion criteria and critically assessed the quality of the studies.

**Results:**

18 studies met our inclusion criteria. The review identified that non-punitive response to error is seen as a serious issue which needs to be improved. Healthcare professionals in the Arab countries tend to think that a ‘culture of blame’ still exists that prevents them from reporting incidents. We found an overall similarity between the reported composite score for dimension of teamwork within units in all of the reviewed studies. Teamwork within units was found to be better than teamwork across hospital units. All of the reviewed studies reported that organisational learning and continuous improvement was satisfactory as the average score of this dimension for all studies was 73.2%. Moreover, the review found that communication openness seems to be a concerning issue for healthcare professionals in the Arab countries.

**Conclusions:**

There is a need to promote patient safety culture as a strategy for improving the patient safety in the Arab world. Improving patient safety culture should include all stakeholders, like policymakers, healthcare providers and those responsible for medical education. This review was limited only to English language publications. The varied settings in which the HSPSC was used may have influenced the areas of strengths and weaknesses as healthcare workers' perception of safety culture may differ.

**Strengths and limitations of this study**

- The first systematic review carried out to report on the status of patient safety culture in Arab countries.
- The first systematic review to report on the use of the Hospital Survey on Patient Safety Culture in Arab countries.
- The review provides a comprehensive insight into the areas of strength and areas of improvement in relation to patient safety culture in Arab countries.
- Owing to the lack of relevant research literature in Arabic, this systematic review was restricted to English language publications only.
- The reported studies were conducted in different healthcare settings (eg, secondary and primary care) which might influence the perception of patient safety culture among healthcare workers.

## Introduction

For decades, human errors in complex systems have been a topic of debate due to their consequences. For example, system failures in the aviation industry cause great publicity and are addressed very quickly because of the impact they have as they involve a large number of people and resources. In the healthcare sector, accidents can be deadly, but they do not attract as much attention as they affect fewer people at a time. Errors in healthcare settings began to draw public attention since the 1990s.^1^ ‘To err is human’ has triggered debate on the important issue of patient safety and a commitment to deliver high-quality, safe healthcare has become a policy goal of governments around the world over the past 15 years.^2^ However, patients are continuing to suffer avoidable harm and substandard care.^3^ According to the Institute of Medicine, safety is defined as ‘the freedom of accidental injury’. On the other hand, the WHO defines patient safety as the ‘reduction of risk of unnecessary harm associated with healthcare to an acceptable minimum’. Safety culture has now become a significant concept for healthcare organisations that are determined to improve patient safety. It is very important that the dynamics of this construct ‘safety culture’ are understood to produce positive change. Hence, it is vital that the underlying cultural factors present within an organisation should be known, so that the safety culture can be transformed.^4^ Several studies have investigated this area at organisational and individual levels in relation to safety culture with different measurements, techniques and modes, so that the contributing factors are understood. Thus, managers and leaders are encouraged to establish and sustain safety culture, as it is fundamental to total systems safety.^5^

Patient safety is based on continuous learning as there is a great need to report and learn from errors, accidents, near misses and adverse events, so that such events can be avoided in the future. The traditional approach to patient safety which is based on establishing mortality committees and scrutinising accidents^6^ can no longer be efficient as the healthcare system is becoming more complex.^7^ Major health organisations such as the WHO, National Patient Safety Foundation (NPSF), the Joint Commission International (JCI) and the Institute for Health Care Improvement (IHI) are encouraging healthcare organisations to develop a culture of safety as an effective strategy for sustainable safety improvement.^8^ A growing body of evidence indicates that the rate of medical errors and adverse events are associated with the attitudes of healthcare professionals towards safety.^9^ In this regard, patient safety culture, which is considered as a component of the organisational culture, includes the shared beliefs, attitudes, values, norms and behavioural characteristics of employees^10^ which will consequently influence the staff member attitudes and behaviours with regard to their organisation's ongoing patient safety performance. Frameworks, surveys and assessment tools have been developed over the past decade to help organisations measure and understand what type of culture exists in the organisation and also to identify areas of strength and gaps, so that factors that might improve or hinder improvement efforts can be identified.^4^ ^11^ ^12^

### Patient safety in the Arab countries

The developed and developing world are aiming to improve patient safety.^13^ Developing countries have been encouraged by the joint global initiative of the WHO and the World Alliance for Patient Safety (WAPS) to launch a concerted effort which will help in the assessment of the magnitude of the problem. One of the studies that evaluated patient safety in Arab countries has used the Patient Safety Friendly Hospital Initiative (PSFHI) standards. PSFHI is one of the initiatives of WHO that helps in supporting institutions in countries to launch a comprehensive patient safety programme, which was launched by the Eastern Mediterranean Regional Office of the WHO in 2007 to help improve the level of patient safety in the region.^14^ Ministries of health in seven developing countries have been assessed using the PSFHI which included Egypt, Jordan, Morocco, Pakistan, Sudan, Tunisia and Yemen. One hospital in each country was assessed against the PSFHI standards. The study found that none of the participating hospitals achieved a baseline score of 50% across the PSFHI standards. It was also found that commitment of the leadership and management are some of the key areas that are wanting in all institutions. Leadership is significant in patient safety as it recognises safe care as a system-related problem.^15^ Furthermore, it was found that patients in these countries are not being involved nor do they have a ‘voice’ in matters related to the care they receive. Another study which was carried out by the WHO^16^ found that no accreditation programmes are being adopted in the Eastern Mediterranean region. This has encouraged few countries in the region to develop and implement accreditation programmes to healthcare institutions.^17^ More importantly, it was reported that accreditation programmes have helped in improving the perception on the quality of patient care and patient safety in Saudi hospitals.^18^ Furthermore, a study was performed in hospitals in Egypt, Jordan, Kenya, Morocco, South Africa, Sudan, Tunisia and Yemen to assess the frequency and nature of adverse events to patients of these countries. The study found that of the 15 548 records that were reviewed, 8.2% showed at least 1 adverse event. A range of 2.5–18.4% per country was found and 83% of these adverse events were judged to be preventable. The study also found that 30% of these events were associated with the death of the patient.^19^ It was reported that one in seven patients suffers harm in Palestinian hospitals.^20^ These statistics suggest that patient safety is a major concern for the health policy agenda in Arab countries and it is vital that the causes of harm to patients are identified and understood to develop strategies for improvement.

### The Hospital Survey on Patient Safety Culture

One of the widely used and validated tools for measuring patient safety culture is the Hospital Survey on Patient Safety Culture (HSPSC) which was developed by the Agency for Healthcare Research and Quality (AHRQ) in the USA.^21^ This instrument consists of 12 dimensions as shown in online supplementary appendix A where each dimension consists of 3–4 survey items, totalling 42 survey items. The survey uses a five-point Likert response scale of agreement ‘Strongly disagree to strongly agree’ or frequency ‘Never to Always’. The survey has been widely used in many countries worldwide, some of these are highlighted in online supplementary appendix B. The aim of this systematic review is to identify the overall status of patient safety culture in the Arab countries. The review also aims to explore the weaknesses, strengths and future opportunities for patient safety improvement in the Arab region.

10.1136/bmjopen-2016-013487.supp1 supplementary appendix

10.1136/bmjopen-2016-013487.supp2 supplementary appendix

## Methods

### Search strategy

Electronic search of MEDLINE, EMBASE, CINAHL, ProQuest and PsychINFO, Google Scholar and PubMed databases, with manual searches of bibliographies of included articles and key journals. English and Arabic language studies published between January 2005 and December 2015 that used the HSPSC in a healthcare setting in any Arab country. Table 1 summarises the keywords that we used to execute the search.

**Table 1:** Summary of the search terms

Field	Database
Arab countries	*Mesh terms*: Arab, Arab countries, Arab world*All fields*: Algeria or Bahrain or Egypt or Iraq or Jordan or Kuwait or Lebanon or Libya or Mauritania or Morocco or Oman or Palestine or Qatar or Saudi Arabia or Sudan or Syria or Tunisia or United Arab Emirates (UAE) or Yemen, or Arabic, or middle east
Safety	Safety or patient safety or patient culture or hospital safety or healthcare safety or safety climate
Healthcare setting and participants	Hospital or nursing or healthcare worker or teaching hospital or primary healthcare, or clinic or government hospital or private hospital
Assessment terms	Perception or assess or measure
Survey instrument	Hospital survey on patient safety culture

### Inclusion and exclusion criteria

Titles and abstracts were examined independently by two reviewers (ME and AA) and were selected or excluded based on the following criteria:

*Selection criteria*: we included studies if they met the following criteria: (1) were concerned with any of the Arab countries mentioned in table 1, (2) used the HSPSC as an instrument for assessing patient safety culture, (3) were published in English or Arabic language and (4) were conducted in primary care, secondary or tertiary care settings.

**Figure 1:**
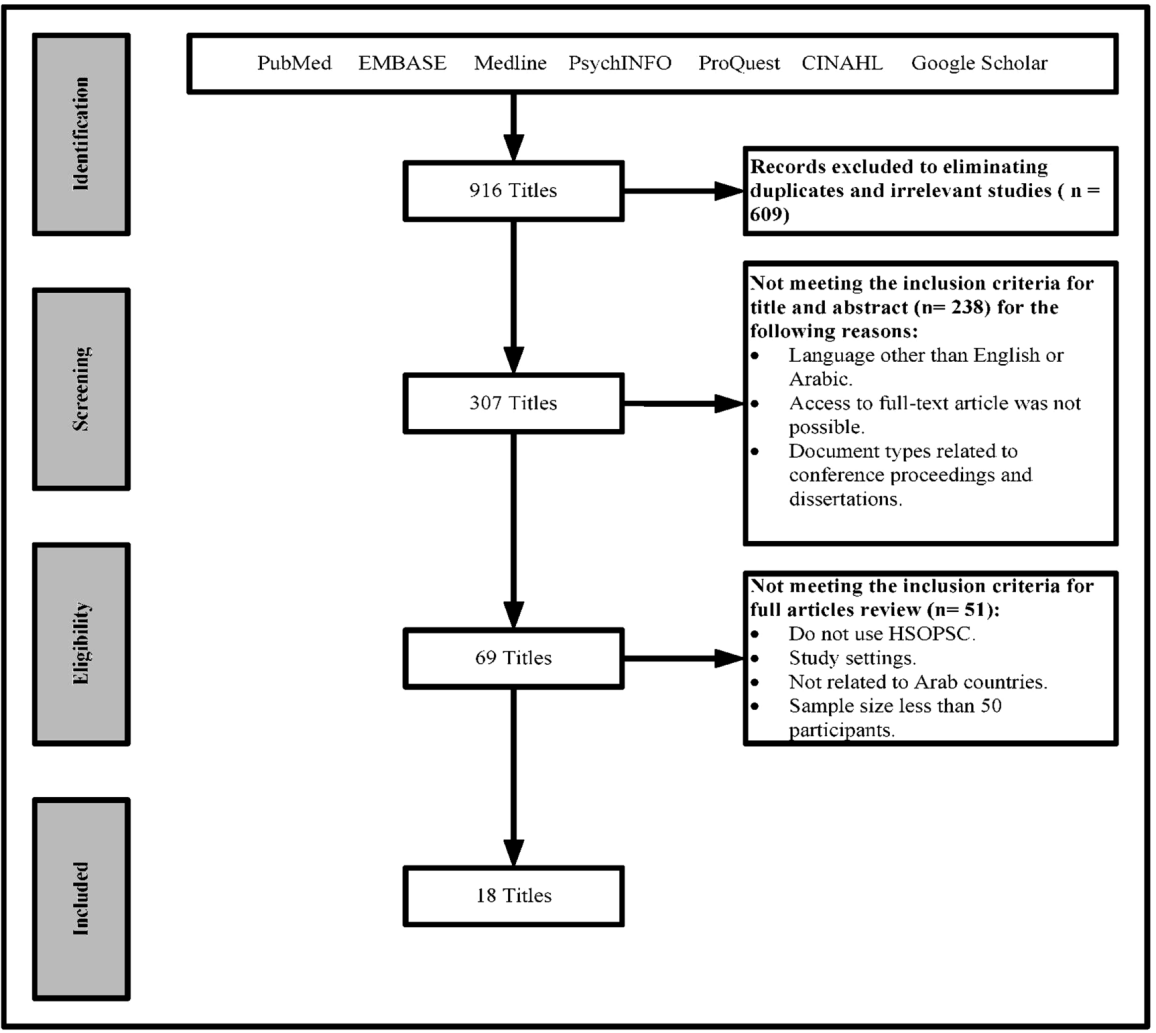
Selection process workflow.

*Exclusion criteria*: we excluded studies based on the following criteria: (1) studies that use tools other than the AHQR's HSPSC, (2) studies that are conducted in residential care facilities, (3) studies that involve patients, (4) studies that are conducted in non-Arab countries and (5) studies that have <50 participants. The flow chart in figure 1 illustrates our selection process.

### Data extraction

We extracted data related to study setting, type and number of participants, composite scores for individual HSPSC categories and origin of the study.

### Quality appraisal

We assessed the quality of included studies in our review through an adapted version of the Newcastle–Ottawa scale.^36^ The assessment can be found in online supplementary appendix C.

10.1136/bmjopen-2016-013487.supp3 supplementary appendix

## Results

Eighteen papers met the inclusion criteria. The country with the most studies was Saudi Arabia (6 studies), while the rest were distributed as follows: Egypt (4 studies), Jordan (3 studies), Oman (2 studies), Kuwait (1 study), Lebanon (1 study) and Palestine (1 study). Of the 18 papers, 16 were conducted among hospital staff, while 2 were performed in a primary care setting. Collectively, the studies include a total of 17 541 participants. Most selected studies had a response rate of above 60% except Ahmed *et al*,^37^ AbuAlRub *et al*,^38^ El-Jardali *et al*^34^ and Alahmadi^35^ which had a response rate of 59.2%, 57%, 55.5% and 47.7%, respectively. Only two studies used electronic and paper format to collect data, whereas the rest of the studies used paper format only.

### Type of participants

We observed a wide variation in the type of participants in our reviewed studies. The majority of the studies have included multiple healthcare professionals. Out of these studies, seven have included non-clinical staff (eg, administrative, managerial staff). Seven studies have surveyed only nurses.

### Strengths and area of potential improvement

Participants in the studies differed significantly in their positive response to different items in the survey. Surprisingly, there was a recurring theme among studies regarding perceptions of patient safety culture. Participants perceived non-punitive response to error to be the least practised in their organisation (table 2). This composite is defined as the extent to which staff feel that their mistakes and event reports are not held against them and that mistakes are not mentioned in their personnel files. On the other hand, organisation learning and continuous improvement were positively rated along with teamwork within units in most of the studies. Table 3 summarises the key strengths and areas of potential improvement based on each country; only three areas of strength and three areas that require improvements have been added in the table as these have received the highest and lowest ranking, respectively.

**Table 2:** Per cent of positive score in each dimension

		Safety culture dimensions (percentage of positive score)*											
First author, year	Country	1	2	3	4	5	6	7	8	9	10	11	12
Aboul-Fotouh, 2012^40^	Egypt	34.6	39.7	33.4	24.6	27.2	19.5	78.2	33.9	49.3	46.4	38	58.1
AbuAlRub, 2014^38^	Jordan	60.3	75	64.3	33.5	70.3	26.2	84.8	60.6	32.9	56.5	57.3	83.8
El-Jardali, 2010^34^	Lebanon	57.3	68.1	68.2	49.7	78.4	24.3	78.3	72.5	36.8	66.4	56.0	82.3
Alahmadi, 2010^35^	Saudi Arabia	60	77	63	61	74	22	87	59	27	70	50	84
El-Jardali, 2014^43^	Saudi Arabia	42.9	63.3	59.4	51.5	70.4	26.8	79.6	65.3	35.1	60.6	61.6	78.5
Al-Ahmadi, 2009^44^	Saudi Arabia	44.2	63.3	56.2	47.6	65.4	21.1	75.9	51.4	31.2	64	56.3	69.9
Al Awa, 2012^18^	Saudi Arabia	36	58	57	47	61	16	74	45	15	51	51	68
Ammouri, 2014^45^	Oman	49.7	68.7	58.8	57.7	25.2	21.4	81.1	50.7	27	60	66.1	83.4
Hamdan, 2013^39^	Palestine	36	46	35	48	37	17	62	43	38	56	44	71
Abdelhai, 2012^46^	Egypt	38	63	43.5	57	57.3	29.9	50	71.1	40	35.5	41.1	51.7
Ahmed, 2011^37^	Egypt	37.9	35.4	33.7	52.1	56.3	52.9	57.1	56.5	42.9	59.6	61.8	59.6
Saleh, 2015^47^	Jordan	46.1	46	37	44.3	44.5	30.7	49.2	43.3	30.4	43.3	43.8	49.8
Khater, 2014^48^	Jordan	49	59.5	69.1	41.7	53.5	21	68.1	60	34.5	57.9	41.7	78.8
Ghobashi, 2014^41^	Kuwait	45	62	32	47	67	24	75	61	41	53	63	82
Aboshaiqah, 2013^49^	Saudi Arabia	36	67	61	22	90	49	82	52	54	49	55	70
Aljabri, 2012^50^	Saudi Arabia	51	71	57	51	73	22	79	57	31	67	60	77
Al-Mandhari, 2014^51^	Oman	54	62	65	44	67	25	84	53	30	60	64	83
Mohamed, 2015^42^	Egypt	66.7	66.7	60	75	80	66.7	73.3	60	60	75	70	80

*1, communication openness; 2, feedback and communication about error; 3, frequency of events reported; 4, handoffs and transitions; 5, management support for patient safety; 6, non-punitive response to error; 7, organisational learning—continuous improvement; 8, overall perceptions of patient safety; 9, staffing; 10, supervisor/manager expectations and actions promoting safety; 11, teamwork across units; 12, teamwork within units.

**Table 3:** Summary of the reviewed studies

No	First author, year	Country	Setting	Number of participants	Participants type	Strength	Area of potential improvement
1	Aboul-Fotouh, 2012^40^	Egypt	1 teaching hospital	510	Health professional	Organisational learning/continuous improvement Teamwork within unit Staffing	Non-punitive response to error Handoffs and transition Hospital management support for patient safety
2	AbuAlRub, 2014^38^	Jordan	1 hospital	57	Nurses	Organisational learning/continuous improvement Teamwork within unit Feedback and communication about errors	Non-punitive response to error Staffing Handoffs and transition
3	El-Jardali, 2010^34^	Lebanon	68 hospitals	6807	Clinical and non-clinical staff	Teamwork within unit Hospital management support for patient safety Organisational learning/continuous improvement	Non-punitive response to error Staffing Hospital handoffs and transitions
4	Alahmadi, 2010^35^	Saudi Arabia	13 hospitals	223	Health professionals	Organisational learning/continuous improvement Teamwork within unit Feedback and communication about errors	Non-punitive response to error Staffing Teamwork across hospital units
5	El-Jardali, 2014^43^	Saudi Arabia	1 hospital	2572	Clinical and non-clinical staff	Organisational learning/continuous improvement Teamwork within unit Hospital management support for patient safety	Non-punitive response to error Staffing Communication openness
6	Al-Ahmadi, 2009^44^	Saudi Arabia	9 public hospitals and 2 private	1224	Clinical and non-clinical staff	Organisational learning/continuous improvement Teamwork across hospital units. Supervisor/manager expectations and action promoting safety	Handoffs and transition Communication openness Non-punitive response to error
7	Al Awa, 2012^18^	Saudi Arabia	1 hospital	605	Nurses	Organisational learning/continuous improvement Teamwork within unit Hospital management support for patient safety	Non-punitive response to error Staffing Communication openness
8	Ammouri, 2014^45^	Oman	4 hospitals	414	Nurses	Teamwork within unit Organisational learning/continuous improvement Feedback and communication about errors	Non-punitive response to error Hospital management support for patient safety Staffing
9	Hamdan, 2013^39^	Palestine	11 hospitals	1460	Clinical and non-clinical staff	Teamwork within unit Organisational learning/continuous improvement Supervisor/manager expectations and action promoting safety	Non-punitive response to error Frequency of events reported Communication openness
10	Abdelhai, 2012^46^	Egypt	Teaching hospitals	400	Health professionals	Feedback and communication about error Hospital management support for patient safety Hospital handoffs and transitions	Non-punitive response to error Supervisor/manager expectations and action promoting safety Staffing
11	Ahmed, 2011^37^	Egypt	2 university hospitals	128	Nurses	Teamwork across hospital units Supervisor/manager expectation and actions promoting safety Teamwork within units	Frequency of reported events Feedback and communication about error Communication openness
12	Saleh, 2015^47^	Jordan	2 public hospitals, 2 private hospitals and 1 teaching hospital	242	Nurses	Teamwork within units Organisational learning/continuous improvement Communication openness	Staffing Non-punitive response to error Supervisor/manager expectations and actions promoting safety
13	Khater, 2014^48^	Jordan	15 public hospitals, 4 privates hospitals and 2 teaching hospitals	658	Nurses	Teamwork within units Organisational learning/continuous improvement Frequency of events reported	Non-punitive response to error Staffing Handoffs and transitions
14	Ghobashi, 2014^41^	Kuwait	4 primary care centres	369	Clinical and non-clinical	Teamwork within units Organisational learning/continuous improvement Management support for patient safety	Non-punitive response to error Frequency of events reported Staffing
15	Aboshaiqah, 2013^49^	Saudi Arabia	1 hospital	498	Nurses	Hospital management support for patient safety Organisational learning/continuous improvement Teamwork within units	Hospital handoffs and transitions Communication openness Non-punitive response to error
16	Aljabri, 2012^50^	Saudi Arabia	2 hospitals	726	Health professionals	Organisational learning/continuous improvement Teamwork within units Hospital management support for patient safety	Non-punitive response to error Staffing Hospital handoffs and transitions
17	Al-Mandhari, 2014^51^	Oman	5 hospitals	398	Clinical and non-clinical	Organisational learning/continuous improvement Teamwork within units Management support for patient safety	Non-punitive response to error Staffing Handoffs and transitions
18	Mohamed, 2015^42^	Egypt	28 primary health centres	250	Clinical and non-clinical	Teamwork within units Management support for patient safety Supervisor expectations and actions promoting safety	Staffing Frequency of events reported Non-punitive response to errors

As noted in table 3, the dimensions that were rated as strengths included organisational learning/continuous improvement, teamwork within units and hospital management support for patient safety. On the other hand, non-punitive response to error, staffing and communication openness were seen as areas that require further improvement in the perception of the participants in these studies. As seen in table 2, most of the studies reported that non-punitive response to error is the least positively ranked area irrespective of the type of the participants or the study sittings. The lowest mean score of the non-punitive response to error dimension was scored in Saudi Arabia,^18^ Palestine^39^ and Egypt^40^ (16%, 17% and 19.5%, respectively). Studies that were conducted in primary care settings^41^ ^42^ did not show any variation in terms of positive scores on the organisational learning and continuous improvement dimension when compared with the studies carried out in hospital settings as well as on the dimension of teamwork within units.

## Discussion

This review has focused on the status of patient safety culture in Arab countries to identify the areas of strength and the areas of concerns. Since the publication of the Institute of Medicine (IOM) report ‘To err is human’,^2^ patient safety culture has become a core element in improving patient safety. It was suggested in the IOM's report that any efforts which aim to improve patient safety culture should move away from a ‘blame culture’ and focus on removing ‘error provoking’ aspects of care delivery systems.^2^ The review showed that healthcare organisations in the Arab world are moving towards the need to assess and evaluate the patient safety culture of healthcare professionals to ensure that improvement strategies are being developed on evidence-based practices. It could also be argued that policymakers and practitioners in developing countries are becoming more aware of the risk of unsafe healthcare.^19^ ^52^ The review showed that HSPSC is not widely used in the Arab world as HSPSC was only used in Saudi Arabia, Egypt, Jordan, Oman, Lebanon, Kuwait and Palestine. None of the reviewed studies were conducted in Arab countries that are located in North Africa. Interestingly, the English and Arabic versions of the HSPSC questionnaire were used in the selected studies. Eight studies have used the Arabic version, but only one study reported that the psychometric properties of Arabic version were validated.^34^

It was clear that healthcare staff in the Arab world are concerned about having a supportive organisational structure that encourages error reporting. There is growing evidence that suggests that the rate of medical errors and adverse events is associated with the attitudes and perceptions of professionals towards safety.^9^ ^53^ Moreover, a study by Najjar *et al*^54^ which looked at the relationship between patient safety culture and adverse event rates in Palestinian hospitals found that departments with positive patient safety culture had lower rates of adverse events. In this regard, many developed countries have realised the importance of improving personnel safety behaviours to achieve high safety and reliability in addition to exploiting modern technology and advanced managerial systems.^55^ Non-punitive response to error has been negatively rated by all participants in all countries which suggests that a culture of medical dominance exists in the organisational structures of the Arab health systems. This medical dominance will also have influence on the interprofessional relationships between the healthcare staff as argued by Adamson *et al*.^56^ The success or failure of team-based work systems implementation has been linked to effective leadership which is a key variable for the functioning of teams.^57^ ^58^ To ensure safe and efficient work medical teams, interactive human factors such as communication, supervision or team structure were considered vital to achieve that.^59^ ^60^ On the other hand, lack of coordinated care or team work failure and breakdowns in communication will result in an unfavourable outcome for the patient.^53^ There is a need to encourage team members through positive behaviour and feedback by leaders.^61^ Ensuring accountability with avoiding blame and negativity needs to be balanced by the leaders.

### System changes to improve patient safety

Patients have a right to be protected by healthcare providers.^62^ Systems that minimise the likelihood of errors while maximising the likelihood of intercepting these errors need to be established.^63^ An efficient operating mechanism that is dependent on the collaboration of the subparts to achieve an outcome is what describes the system approach to patient safety.^64^ Provider organisations across different care settings, policymakers, regulators, educational institutions and patients are the subparts of the patient safety system.^64^ The rate of errors in complex systems such as those that deliver healthcare is influenced by many factors. A nested hierarchy of factors that determine the safety of a healthcare system have been suggested by Vincent *et al*.^65^ The system factors related to work environment, institutional context, organisation and management, individual team members, tasks and patients.^65^ This could imply that improving work conditions would involve improvements to interface design, to the physical environment, to the ergonomics of equipment or the reduction in distractions and interruptions which influence the propensity to error.^7^ A basic requirement of a safe system is using protocols, checklists and other reminders for patient and clinician interactions.^7^ The use of these aids would benefit making informed decisions and creating a culture of safety through complying with rules and procedures. A widely known and well-established systems-based model in patient safety research is known as ‘Swiss Cheese Model’ of safety^66^ in which it proposed that hazards within complex systems are prevented by a series of barriers. Thus, a systems-based approach to patient safety in Arab countries is highly needed.

### Patient safety reporting systems

The review indicated that healthcare staff in the Arab world are not being encouraged to report incidents which can be investigated, so that lessons can be learnt. This could be because such reporting systems are not being implemented by healthcare organisations in the region due to the lack of the required regulations to manage and promote patient safety as the case in developed countries, including the USA.^67^ Hence, as argued by Leape,^68^ adverse event-reporting systems will not be efficient within a punitive culture. Thus, there is a need for a fundamental culture change to ensure that all innovations which are introduced to improve patient safety can achieve their potential. More importantly, healthcare staff should have the opportunity to learn from such reporting systems by ensuring that there is a policy for effective feedback as that will help organisations learn from failures in the delivery of care.^69^ In other words, policymakers should work in collaboration with healthcare providers to ensure that reporting systems are being implemented and that staff are encouraged and supported to report any incidents which might have an impact on the patient's health. Leape^70^ affirmed that patient safety reporting systems helps healthcare organisations to improve patient safety which is very significant to Arab countries as reported by this review. This is to ensure that when incidents occur in the workplace, organisations need to understand what happened and why so that the probability of recurrence is reduced and to figure out if the existing interventions are effective or not.^71^

### ‘Just’ culture

A prerequisite to achieving lasting improvement is the need to embed the goal of providing safe care in the culture of the organisation.^72^ Culture transformation is a complex endeavour as it evolves in response to past events, local conditions, the mood of the workforce and the character of the leadership.^73^ On the other hand, safety culture reflects the ‘ability of individuals or organisations to deal with risks and hazards so as to avoid damage or losses and yet still achieve their goals’.^66^ Thus, organisations need to ensure that a positive patient safety culture is in place which encourages honest disclosure of information and that a sincere interest in rectifying the problems is demonstrated.^72^ It could also be assumed that healthcare organisations in the Arab world should move away from the culture of blame to a ‘just’ culture which encourages the acknowledgement of error so that learning from errors can be achieved. In other words, employees need to feel that they will not be punished or concealed for acknowledging errors. This could imply that positive safety culture will help in encouraging honesty and fostering learning by balancing the individual and organisational accountability to achieve better quality care.

### Education and regulation

Milligan^74^ pointed out that a more fundamental change is required within the healthcare curricular in order to improve patient safety. This suggests that countries in the Arab world should focus on the need of providing training and education programmes to healthcare professionals and students on the importance of systems approach in creating a patient safety culture. The review indicated that organisational learning and continuous improvement has been ranked as one of the areas of strength in the Arab countries. However, there needs to be a holistic approach to patient safety management by ensuring that all relevant elements such as resources, training, education and policies are in place to create a sustainable culture of safety in the workplace. In the USA as a result of the IOM's report, the US Congress passed the Patient Safety and Quality Improvement Act in 2005 which aims at improving quality and safety through the collection and analysis of data on patient events. This shows that patient safety has to be improved by the involvement of all relevant stakeholders, including policymakers, healthcare providers and patients as well as their families.

### Communication and staffing

The review indicated that staff in the healthcare sector in the Arab world perceived teamwork within units to be acceptable when compared with other dimensions of the survey. Teamwork efforts cannot be successful without open communication within the care team. Thus, it is vital that training programmes on teamwork and communication are provided to all staff, so that different disciplines in medicine are connected to improve team performance. It was revealed by Pronovost *et al*^75^ that the Accreditation Council on Graduate Medical Education in the USA endorsed the inclusion of ‘interpersonal and communication skills’ in their core competencies in 1999. This shows that establishing interdisciplinary team training can help in improving patient safety as it will help in reducing the misunderstandings among individuals and teams which might have impact on the patient's care. Therefore, policymakers in the Arab countries should ensure that staff have the ability to speak up when there are concerns in the workplace as well as being able to consult and seeking help whenever it is needed to ensure high-quality care is provided to all patients at all times. It was also found that level of staffing is a common issue that is shared by many Arab countries. Insufficient staffing is likely to cause stress as staff will be forced to work under pressure and for long hours. It is well evidenced that when nurses work more than 12 hours at one time, the risk of making an error increases significantly.^76^

### Limitations

Owing to the lack of relevant research literature in Arabic, this systematic review was restricted to English language publications only that have been peer-reviewed. This could have limited other studies that have used the HSPSC. The reported studies were conducted in different healthcare settings (eg, secondary and primary care) which might influence the perception of patient safety culture among healthcare workers. In terms of the limitations of the review, responses to patients' safety culture items are likely to be dependent on the organisation where the interviews have been conducted. Intrinsic organisational culture elements such as management support, level of funding and size of the organisation can limit the generalisability of some of the reviewed studies. Another limitation is the fact that not all Arab countries have used the HSPSC which could also limit the generalisability of the reviewed studies. Moreover, the comparison between the reviewed studies may be difficult because of the variation in the type of participants (eg, nurses, doctors and administrators). Nurses may have a different perception of patient safety culture as they are in continuous contact with patients. We argue that mixed-method studies can help in enhancing the understanding of patient safety culture in the healthcare sector in Arab countries. The use of qualitative approaches such as semistructured interviews and focus groups with healthcare professionals, patients, policymakers and familiar can further identify the root causes of poor safety culture that exist in some healthcare settings. Qualitative findings can support quantitative findings and provide more insightful explanations about the challenges that face health systems in Arab countries.

## Conclusion

Patient safety remains a global problem that affects the developed and developing countries. Healthcare organisations should focus on the need of assessing safety culture as that will provide basic understanding of the safety-related perceptions of their staff. Safety culture assessment tools can help healthcare organisations in identifying the areas for improvement. It is also very important that policymakers in the Arab countries establish a just culture in the workplace where employees should be encouraged to report any adverse events, errors, incidents or near misses so that lessons can be learnt. More importantly, safety culture should be assessed on a regular basis to evaluate the effectiveness of patient safety programmes and interventions. Healthcare leaders, researchers and legislators in the Arab countries need to realise that patient safety is a serious public health concern which costs lives. This review has identified that non-punitive response to error is seen as a serious issue which needs to be improved as healthcare professionals in the Arab countries tend to think that a ‘culture of blame’ still exists that prevents them from reporting incidents. Thus, policymakers need to ensure that legislations and regulations are introduced to encourage healthcare organisations to implement patient safety reporting systems which will help identify risks to patients and help healthcare organisations learn from their mistakes.
