# Supplementary material for: Status of patient safety culture in Arab countries: a systematic review
Source: BMJ Open. 2017 Feb 24;7(2):e013487. doi: 10.1136/bmjopen-2016-013487 (PMC5337746; doi:10.1136/bmjopen-2016-013487)
Supplement: supplementary appendix [file bmjopen-2016-013487supp_appendixA.pdf]

Appendix A. Patient safety aspects in HSPSC

**Unit-level aspect of safety culture**
Supervisor/Manager Expectations & Actions Promoting Safety (4 items)<br>Organisational Learning - Continuous Improvement (3 items)<br>Teamwork Within Units (4 items)<br>Communication Openness (3 items)<br>Feedback and Communication About Error (3 items)<br>Non-punitive Response to Error (3 items)<br>Staffing (4 items)
**Hospital-level aspects of safety culture**
Hospital Management Support for Patient Safety (3 items)<br>Teamwork Across Hospital Units (4 items)<br>Hospital Handoffs and Transitions (4 items)
**Outcome variables**
Overall Perceptions of Safety (4 items)<br>Frequency of Event Reporting (3 items)
