# Supplementary material for: Status of patient safety culture in Arab countries: a systematic review
Source: BMJ Open. 2017 Feb 24;7(2):e013487. doi: 10.1136/bmjopen-2016-013487 (PMC5337746; doi:10.1136/bmjopen-2016-013487)
Supplement: supplementary appendix [file bmjopen-2016-013487supp_appendixB.pdf]

Appendix B: Some of the countries that have applied the HSPSC

**Author**	**Country**	**Sample Size**
Sorra & Nieva (2004)[21]	USA	1437 (21 hospitals)
Olsen (2008)[22]	Norway	1919clinical/non clinical staff (1 hospital)
Pfeiffer & Manser (2010)[23]	Switzerland	2989 clinical/non clinical staff (1 hospital)
Smits et al. (2008)[24]	Netherlands	583 clinical/non clinical staff (8 hospitals)
Hellings et al. (2007)[25]	Belgium	2813 clinical/non clinical staff (5 hospitals)
Bagnasco et al. (2010)[26]	Italy	724clinical/non clinical
Waterson et al. (2009)[27]	England	1437 clinical/non clinical staff (3 hospitals)
Sarac (2011)[28]	Scotland	1969 clinical staff (7 acute hospitals)
Najjar et al. (2013)[29]	Palestine	2022 clinical/non-clinical (13 hospitals)
Bodur & Filiz2 (2010)[30]	Turkey	309 clinical/non-clinical (3 hospitals)
Robida (2013)[31]	Slovenia	976 clinical/non-clinical (3 hospitals)
Hedsköld et al (2013)[32]	Sweden	84,215 clinical/non-clinical (national-wide survey)
Nie et al. (2013)[33]	China	1160 physicians and nurses (32 hospitals)
El-jardali et al. (2010)[34]	Lebanon	6807 clinical/non-clinical (68 hospitals)
Alahmadi (2010)[35]	Saudi Arabia	223 clinical/non-clinical (13 hospitals)
