# Supplementary material for: Status of patient safety culture in Arab countries: a systematic review
Source: BMJ Open. 2017 Feb 24;7(2):e013487. doi: 10.1136/bmjopen-2016-013487 (PMC5337746; doi:10.1136/bmjopen-2016-013487)
Supplement: supplementary appendix [file bmjopen-2016-013487supp_appendixC.pdf]

APPENDIX C. RESULTS OF THE CRITICAL APPRAISAL OF THE INCLUDED STUDIES.

no	Study (first author), year	**Selection**				**Comparability**	**Outcome**		Total out of 10
		Representativeness of the sample*	Sample size	Non respondents	Ascertainment of exposure		Assessment	Statistical test	
1	Aboul-Fotouh, 2012	+	+	+	++	+	+	+	8
2	AbuAIRub, 2014			+	++	+	+	+	6
3	El- Jardali, 2010	+	+	+	++	++	+	+	9
4	Alahmadi, 2010	+		+	+		+	+	5
5	El-Jardali, 2014	+	+	+	++	+	+	+	8
6	Al-Ahmadi, 2009	+	+	+	+	+	+	+	7
7	Al-Awa, 2012	+	+	+	+	+	+	+	7
8	Ammouri, 2014	+	+		++	+	+	+	7
9	Hamdan, 2013	+	+	+	++	+	+	+	8
10	Abdelhai et al 2012	+	+		+	+	+	+	6
11	Ahmed et al 2011	+		+	+	+	+	+	6
12	Saleh et al 2015	+	+	+	+		+	+	6
13	Khater et al 2014	+	+	+		+	+	+	6
14	Ghobashi et al 2014	+	+	+	++		+	+	7

15	Aboshaiqah, 2013	+	+	+	++		+	+	7
16	Aljabri, 2012	+	+	+	+		+	+	6
17	Al-Mandhari et al 2014	+	+	+	++		+	+	7
18	Mohamed et al 2015	+		+	++		+	+	6
